# Network Pharmacology Study on the Mechanisms of *Panax Notoginseng* in the Treatment of Diabetic Retinopathy and Cataract

**DOI:** 10.1155/abb/6687606

**Published:** 2025-05-11

**Authors:** Ting Zhang, Guangquan Ji, Tianpu Feng, Xi Lin, Lei Wang, Yi Xu, Pan Shi, Wenxue Liang

**Affiliations:** ^1^Department of Clinical Laboratory, The Affiliated Lianyungang Hospital of Xuzhou Medical University (The First People's Hospital of Lianyungang), Lianyungang, Jiangsu, China; ^2^School of Pharmacy, Jinzhou Medical University, Jinzhou, Liaoning, China; ^3^Department of Pharmacy, Xuzhou Medical University Affiliated Hospital (Lianyungang No 1 People's Hospital), Lianyungang, Jiangsu, China; ^4^Animal Center, Kangda College of Nanjing Medical University, Lianyungang, Jiangsu, China

**Keywords:** diabetic cataract, diabetic retinopathy, molecular docking, network pharmacology, *Panax notoginseng*

## Abstract

**Background:** Diabetic retinopathy (DR) and diabetic cataract (DC) are two closely related microvascular complications of diabetes. *Panax notoginseng*, a plant from the Araliaceae family and genus Panax, is widely used in traditional Chinese medicine (TCM) due to its antioxidant, anti-inflammatory, and blood circulation-promoting properties. Recent studies suggest that drugs possessing anti-inflammatory, antioxidant, and blood circulation-promoting characteristics may have unexpected benefits in treating diabetic microvascular complications. This study employs network pharmacology to investigate the mechanisms by which *P. notoginseng* can treat DR and DC as comorbidities.

**Objective:** The study aims to explore the active components and biological mechanisms of *P. notoginseng* in treating these comorbidities using network pharmacology and molecular docking.

**Methods:** Components of *P. notoginseng* were identified through literature reviews and database queries. Active components were selected based on drug-like principles, and their targets were predicted using the principle of similarity. Disease-related genes were collected from OMIM and GeneCards and scored. Venn analysis identified target nodes, followed by protein–protein interaction (PPI) network analysis, gene ontology (GO) analysis, and KEGG pathway analysis. Topological algorithms analyzed the PPI network, and key nodes combined with other analysis results were utilized to construct a *P. notoginseng*-active component-gene-phenotype network using Cytoscape 3.9.1. Molecular docking on key genes, integrated with biological background, determined potential therapeutic targets against the diseases.

**Results:** *P. notoginseng* contains eight active components and 234 potential gene targets. Network analysis showed that *P. notoginseng* can repair microvascular damage by influencing disease-related signaling pathways. Molecular docking indicated that four key targets (SRC, JAK2, IGF1R, and EGFR) effectively bind to the active components of *P. notoginseng*.

**Conclusion:** These findings provide insights into the molecular-level action of *P. notoginseng* against these diseases. Overall, this study enhances our understanding of the potential of *P. notoginseng* in treating DR and DC as comorbidities and establishes a foundation for further research.

## 1. Introduction

Diabetic retinopathy (DR) and diabetic cataract (DC) are among the most common complications in patients with diabetes [1, 2]. Both conditions share common pathogenic mechanisms, particularly relating to damage to ocular microvasculature under hyperglycemic conditions, which frequently coexist in diabetic patients [3, 4]. Damage to ocular microvasculature is often accompanied by chronic inflammation [5], which is closely associated with the development and progression of microvascular complications. Existing research suggests that anti-inflammatory treatments may offer potential benefits in managing glucose levels, reducing microvascular complications, and improving insulin resistance [6].

*Panax notoginseng*, a plant from the Araliaceae family and the Panax genus, has been used in traditional Chinese medicine (TCM) since the Ming Dynasty. Its therapeutic properties were first documented in “The Miraculous Prescription for Traumas” by Zhenren Yiyuan [7] and were further elaborated in Li Shizhen's (Li.,1578) “Compendium of Materia Medica,” where it is described as having hemostatic, blood-scattering, and pain-relieving effects, suitable for treating symptoms such as hemoptysis and epistaxis [8]. The integration of advanced technologies, such as basal insulin therapy (PwT2D-BI) and continuous glucose monitoring (CGM), has shown significant promise in improving glycemic control for individuals with type 2 diabetes (T2D). The role of CGM in the early detection of DR is primarily reflected in real-time blood glucose monitoring, reducing glucose fluctuations, enabling early warning and intervention, supporting personalized treatment, and improving patient compliance to lower the risk of retinopathy, while providing more precise tools and methods for diabetes management. Meanwhile, the role of PwT2D-BI in the early detection of DR is mainly demonstrated through optimizing blood glucose control, reducing glucose fluctuations, enabling early intervention, and providing personalized treatment to lower the risk of retinopathy, while enhancing the precision of diabetes management through machine learning (ML) technology. The two approaches complement each other, jointly offering a scientific and precise solution for the early detection and management of DR.

**Figure 1:**
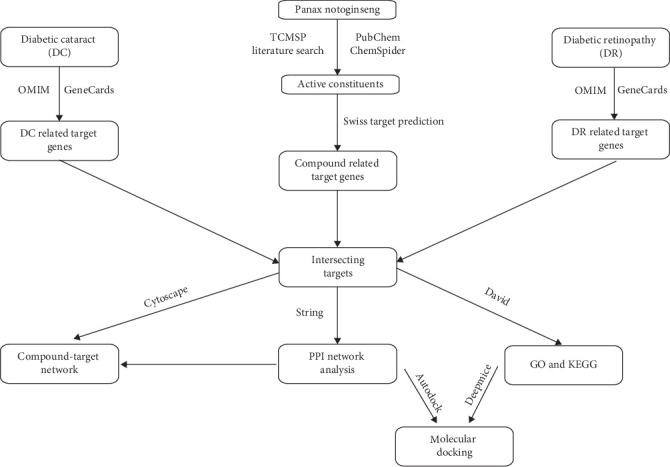
Technical roadmap for the mechanism of notoginsen treating different diseases with a common approach.

This U.S.-based study leverages ML to analyze the effectiveness of these technologies, offering valuable insights into optimizing diabetes management. The results highlight the potential of ML-driven approaches to enhance treatment strategies and patient outcomes [9]. Modern research has demonstrated that *P. notoginseng* possesses anti-inflammatory and antioxidant properties [10, 11]. Several studies have demonstrated that *P. notoginseng* has protective effects against DR. Our research extends these findings by exploring the mechanisms through which *P. notoginseng* can treat both DR and cataracts [12, 13]. Network pharmacology techniques can elucidate the potential of plant-based bioactive compounds [14]. This study utilizes network pharmacology to demonstrate the significant potential of *P. notoginseng* in treating DR and DC, providing a foundation for future pharmacological investigations. A roadmap summarizing the utilized databases, online platforms, and methodologies is presented in Figure 1.

## 2. Methods

### 2.1. Predicting Intersection Targets

#### 2.1.1. Summarizing Active Ingredients of Notoginseng

We conducted a comprehensive literature search using PubMed to compile the phytochemical constituents of notoginseng. Additionally, we utilized the ETCM database and TCMSP database to supplement the phytochemical components of notoginseng. To obtain SMILES and chemical information, as well as the three-dimensional (3D) structures of active ingredients, we utilized the PubChem database. To optimize the assessment of the drug potential of the most promising active ingredients, we considered two factors: oral bioavailability (OB) ≥ 30% and drug-likeness (DL) ≥ 0.18 [15]. These are widely recommended indicators for evaluating absorption, distribution, metabolism, and excretion (ADME) properties. OB refers to the rate and extent to which a drug is absorbed into the bloodstream when administered orally. Meanwhile, DL evaluates the similarity of a drug to known drugs based on physicochemical properties such as solubility, absorption rate, and transformation rate, providing insights into its pharmacokinetic characteristics. Higher OB and DL scores indicate greater drug potential. The databases used are summarized in Table 1.

#### 2.1.2. Prediction of Targets for Active Ingredients of Notoginseng

To gain a deeper understanding of the therapeutic mechanisms of notoginseng, target prediction plays a crucial role. We utilized the SwissTargetPrediction online platform for these predictions. SwissTargetPrediction operates on the “similarity principle,” which suggests that similar molecules tend to have similar properties. In this analysis, we used the SMILES representation of the active ingredients as input and selected humans as the organism of interest. The SMILES codes were obtained from the PubChem database.

#### 2.1.3. Summarizing Potential Targets for Diseases

Identifying disease-related genes is a key step in elucidating the biochemical pathways through which TCM treats various diseases and conditions. In our study, we utilized well-known databases, OMIM and GeneCards, to identify key genes associated with diseases. GeneCards, owing to its extensive data sources and comprehensive information integration, provides a vast amount of gene-related data. In contrast, OMIM focuses on high-quality data concerning genetic diseases through rigorous data curation and detailed descriptions. This distinction leads to notable differences in the size and scope of the two databases. These databases provide valuable information on genes, diseases, and related scientific research. We removed duplicate gene targets obtained from both databases and eliminated targets with weak associations.

### 2.2. Prediction of Hub Genes and Protein–Protein Interaction (PPI) Network

PPIs play a critical role in regulating various biochemical mechanisms and the formation of large molecular complexes. Studying these interactions can provide valuable insights into the mechanisms of drug action, enhancing their efficacy, and minimizing side effects. We submitted the intersecting targets to the STRING database, which provides information on PPIs and allows us to identify functional associations between proteins. To identify hub genes within the network, we utilized the CytoNCA plugin in Cytoscape 3.9.1. Hub genes are those that exhibit high connectivity or interdependence within the network, indicating their crucial roles in the system.

### 2.3. Pathway and Functional Enrichment Analysis

A comprehensive understanding of KEGG pathways and gene ontology (GO) enrichment analysis is essential for gaining insights into the therapeutic effects of drugs against diseases. These analyses involved the pharmacological mechanisms of *P. notoginseng* in combating diseases by examining the involved genes, their functional roles, and the disease-related pathways. This helps us understand how the drug treats specific diseases. In our study, we used the functional enrichment database DAVID for KEGG pathway analysis. We input the common targets of active components and the disease of interest into the DAVID database for the analysis. GO analysis involves classifying genes based on their functions, including three subcategories: molecular function (MF), cellular component (CC), and biological process (BP). We examined and evaluated the top 20 GO results and the most significant KEGG target pathways. To visually represent these KEGG pathways and GO annotations, we created bubble charts using the MicroBioinformatics website. By reviewing existing studies, we visualized the KEGG pathways most closely associated with the disease and highlighted the hub genes identified in our analysis in red.

### 2.4. Construction of Compound Target Network

To explore the potential of notoginseng in disease prevention and gain deeper insights into its therapeutic mechanisms, we employed network analysis. This approach aids in elucidating the therapeutic effects of TCM. To construct the target network, we imported the active components of notoginseng, their associated gene targets, and pathways derived from GO and KEGG analyses of intersection targets into Cytoscape 3.9.1, thereby creating a TCM-active component-hub target-significant pathway-disease network. Cytoscape, a widely used tool in network science, facilitates the visualization of complex networks rich in information. In this network, active components and target genes are represented as nodes, while their interactions are depicted as edges. To further analyze the network and assess its basic topological features, we used network analysis software, which enabled us to calculate key metrics regarding network connectivity and the importance of relationships between active components and their target genes.

### 2.5. Molecular Docking Analysis

3D structures of selected compounds were obtained from the PubChem database, while the protein structures of potential targets were acquired from the RCSB Protein Data Bank (PDB) (http://www.rcsb.org/). Through literature searches, we identified the active sites of the target proteins. High-quality and structurally complete human protein structures were utilized to form docking complexes. To refine the protein structures by relocating ligands and water molecules, we used AutoDOCK software. Furthermore, through literature searches, we determined the active sites of the target proteins. We selected the complex with the highest absolute binding energy for further analysis [16].

## 3. Results

### 3.1. Identification of Intersection Targets

**Figure 2:**
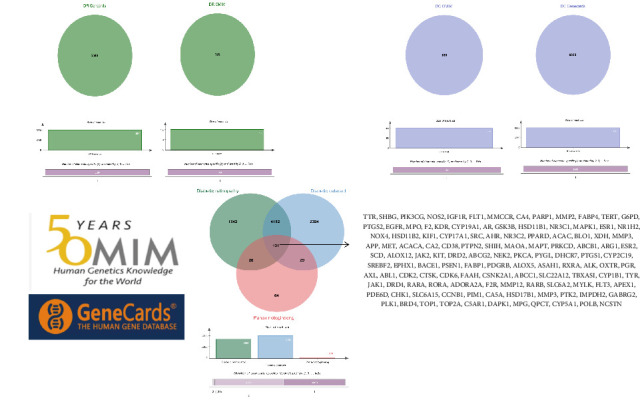
Shows the origin of genes related to diabetic retinopathy (DR), diabetic cataract (DC), the construction of the gene library, and the intersection situation with genes related to notoginseng.

TCMs contain plant compounds with disease-preventive properties. Active components in these medicines exert therapeutic effects on the human body. Comprehensive literature searches and data collection from the TCMSP database identified 119 plant chemical substances related to notoginseng (Supporting Information 2: Table S1). Among these, eight active components met the filtering criteria of DL ≥ 0.18 and OB ≥ 30% (Table 2). Using the Swiss Target Prediction database, we identified 234 targets associated with the eight active substances found in notoginseng. To investigate potential disease-related targets, we utilized the GeneCards and OMIM databases, which yielded 5768 targets for DR and 6846 targets for DC. After removing duplicate entries and integrating disease targets, we ultimately obtained 121 shared targets between notoginseng and disease-related genes (Figure 2). These genes represent the potential targets that were the focus of our research.

### 3.2. PPI Network Analysis

To investigate the PPIs related to diseases, we analyzed the intersection targets using the STRING database and obtained the PPI network (Supporting Information 1: Figure S1). The PPI analysis revealed interactions among various targets, providing valuable insights into the mechanisms of ginseng in treating diseases. The resulting PPI network was topologically analyzed in Cytoscape. To identify hub genes within the PPI network, we utilized the CytoNCA plugin in Cytoscape. Our topological analysis metrics included degree centrality, closeness centrality, betweenness centrality, and eigenvector centrality, with their significance listed in Table 3. We ranked the top 30 targets with the highest scores in Table 4, defining them as hub genes. The score of a gene reflects its connectivity level with other targets, indicating its importance in the PPI network.

### 3.3. GO and KEGG Pathway Analysis

**Figure 3:**
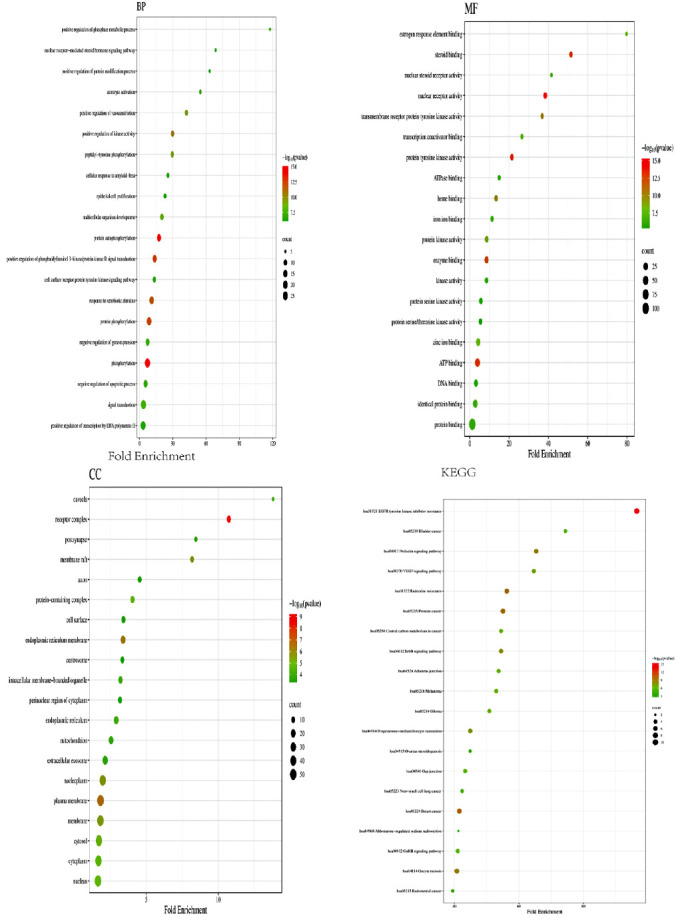
Results of gene ontology (GO) enrichment analysis and dot plot of KEGG pathway enrichment analysis. The horizontal axis represents the gene ratio, while the vertical axis represents the name of the enriched pathway. The color intensity indicates different thresholds of the *p*-value, and the size of the dot indicates the number of genes corresponding to each pathway.

**Figure 4:**
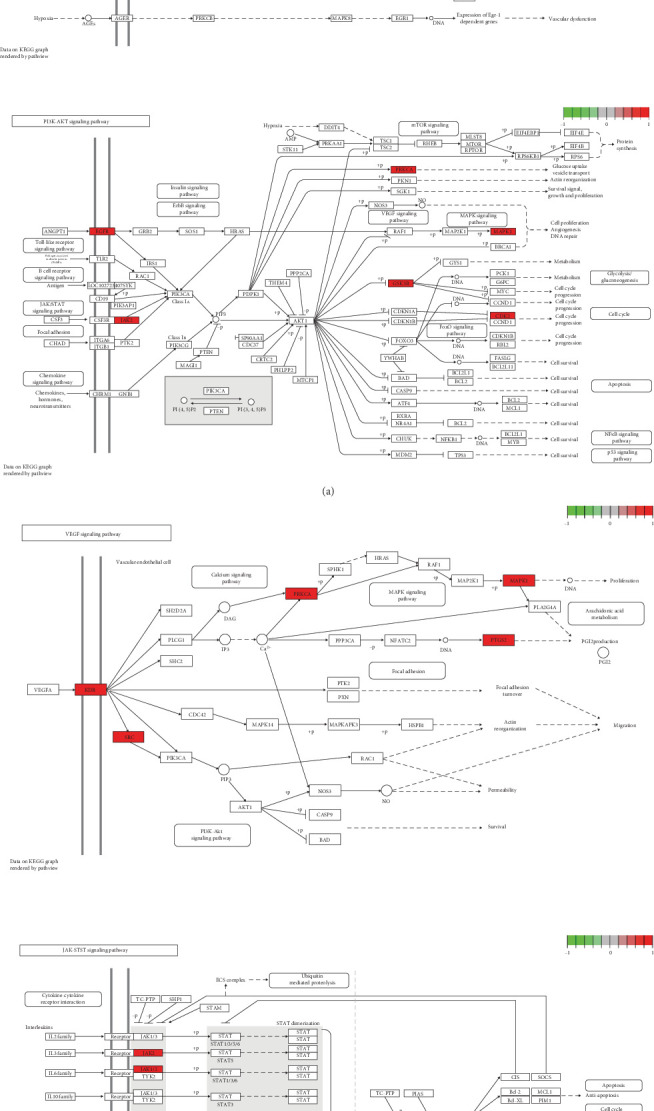
Visualize the KEGG enrichment results and mark the positions of hub genes. (a) Visualization of Age-rage signaling pathway in diabetic complications and PI3K-AKT signaling pathway (hub genes highlighted) and (b) VEGF signaling pathway and JAK-STAT signaling pathway (hub genes highlighted).

Through GO and KEGG pathway analyses, the preliminary biological activities and molecular mechanisms of ginseng targets were elucidated. GO analysis of the intersection targets revealed 410 BPs, including signal transduction and peptide tyrosine phosphorylation, which are highly relevant to the damage of ocular microvasculature under hyperglycemic conditions. Additionally, 67 CCs were identified, such as the cell surface, cell membrane, and endoplasmic reticulum membrane. Furthermore, 106 MFs were recorded, including enzyme binding, transcription factor activity, and protein binding. KEGG analysis predicted 89 pathways associated with the targets. From these pathways, 20 disease-related pathways were selected from the KEGG database. Bubble charts for KEGG pathway and GO analyses, created using the Bioinformatics website (bioinformatics.com.cn), are displayed in Figure 3. By consulting existing research, we visualized the most closely disease-associated KEGG pathways and highlighted the hub genes identified in our analysis in red (Figure 4).

### 3.4. Compound-Target Network Construction

**Figure 5:**
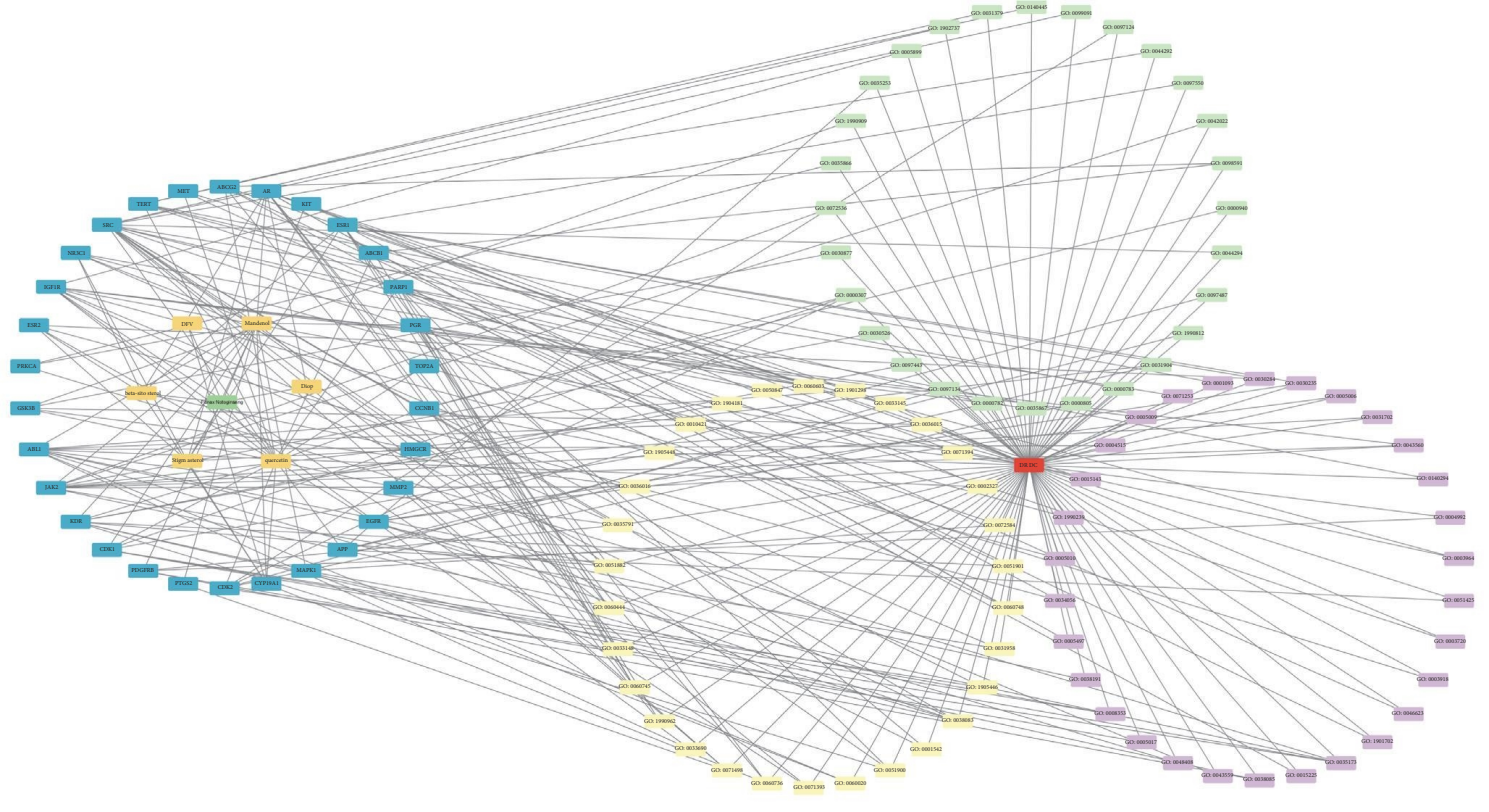
Notoginseng - hub genes - gene ontology:cellular component (GO:CC) - gene ontology:biological process (GO:BP) - gene ontology:molecular function (GO:MF) - KEGG - DR and DC networks.

To better understand the role of *P. notoginseng* (Sanqi) in treating diseases, we constructed an herbal medicine-active component-hub target-significant pathway-disease network using Cytoscape 3.9.2. This network visualizes the relationships between herbal medicine, active components, GO analysis results, significant pathways, genes, and diseases, highlighting their interactions through edge enhancement (Figure 5). To further assess the relative importance of hub genes, we evaluated their connectivity degrees, as shown in Table 5. Among the active components, quercetin achieved the highest connectivity. Based on this analysis, we chose quercetin for more detailed studies using molecular docking technology.

### 3.5. Molecular Docking

**Figure 6:**
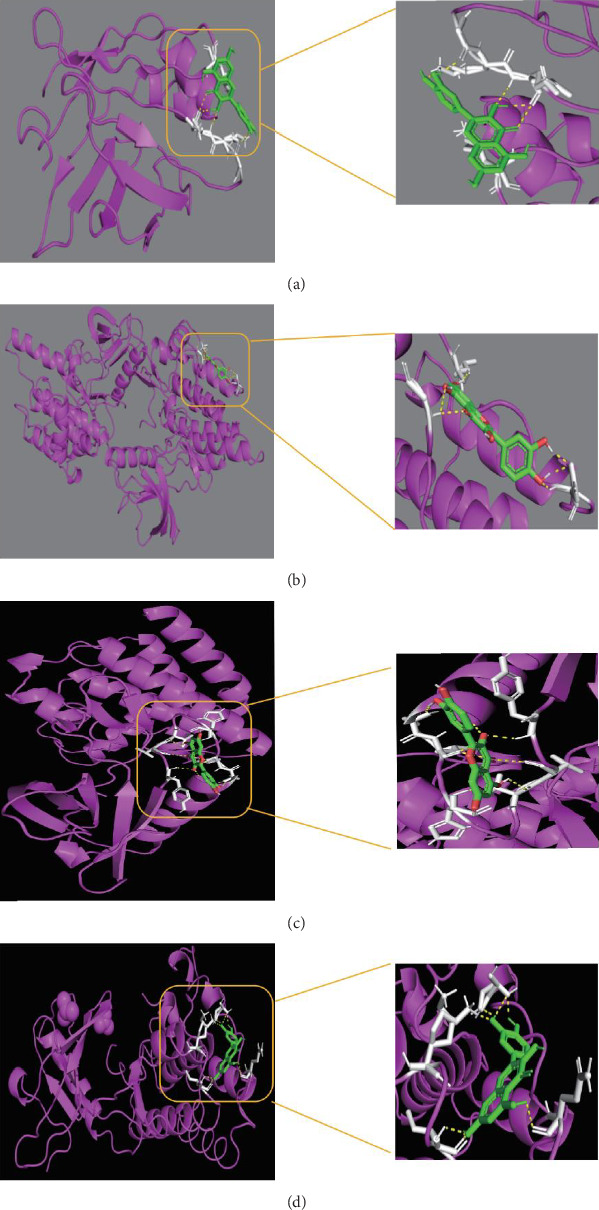
Molecular docking. (a) Molecular docking results of panax notoginseng-derived small molecules with SRC. (b) Molecular docking results of panax notoginseng-derived small molecules with JAK2. (c) Molecular docking results of panax notoginseng-derived small molecules with IGF1R. (d) Molecular docking results of panax notoginseng-derived small molecules with EGFR.

In molecular docking analysis, negative binding energy indicates a tendency for the ligand to form a stable complex with the receptor. Lower energy values signify stronger affinity, thereby enhancing the likelihood of interaction and potential biological effects. By estimating the binding energy in molecular docking, we can model and predict the affinity between ligands and receptors. Following comprehensive research on PPI networks, four key proteins—SRC [22], JAK2 [16], IGF1R [23], and EGFR [24], were selected for molecular docking studies. To perform the docking simulations, the 3D structures of these proteins were obtained from the PDB with the following PDB IDs: SRC (PDB ID: 4BVM), IGF1R (PDB ID: 1P4O), JAK2 (PDB ID: 7TEU), and EGFR (PDB ID: 8A27). The resolutions of SRC, JAK2, IGF1R, and EGFR are 0.93, 1.5, 1.45, and 1.07 Å, respectively. The docking results revealed strong interactions between the compounds and the protein binding pockets (Table 6). Understanding these interactions is crucial for comprehensively understanding how active components influence disease. The docking complexes of the proteins are shown in the Figure 6. Additionally, the docking analysis supports our findings that the active components of ginseng form stable bonds with the targets, further validating their potential therapeutic effects on diseases.

## 4. Discussion

The shared pathogenic mechanisms of DR and DC are associated with microvascular damage caused by diabetes. Long-term hyperglycemia in diabetic patients leads to damage to the ocular microvasculature, triggering both DR and DC. Studies have shown that diabetic patients have an increased risk of developing DR after cataract surgery [25]. Furthermore, cataract surgery itself may accelerate the progression of DR, especially in the short term after surgery [26]. These observations suggest a potential connection between the two diseases.

Specifically, both conditions are closely linked to inflammatory responses and damage to microvasculature. DR results from damage to the microvasculature. Poorly controlled blood glucose levels in diabetic patients lead to damage to the endothelial cells of microvessels, increasing vascular wall permeability. Subsequently, proteins and CCs from the blood leak into the retina, causing inflammation and microvascular lesions. This inflammatory response further exacerbates microvascular damage, leading to retinal ischemia and the formation of new blood vessels, ultimately contributing to the progression of retinal disease [27–29].

The pathogenesis of DC is also linked to inflammation and microvascular lesions. Prolonged hyperglycemia in diabetic patients leads to metabolic disorders in the lens, causing lens proteins to denature and form opacities. Furthermore, diabetes affects the blood supply to the lens, leading to microvascular lesions that exacerbate lens damage. Inflammation plays a significant role in this process; the levels of inflammatory factors such as TNF-α and IL-6 in the serum and aqueous humor of DC patients are significantly higher than those in nondiabetic patients [30]. The elevated levels of these inflammatory factors may result from insulin resistance and hyperglycemia in diabetic patients. Inflammatory factors like TNF-*α* and IL-6 play a crucial role in the development and progression of DCs, possibly by promoting cell proliferation, migration, and recruitment of inflammatory cells, thereby exacerbating damage to ocular tissues [31].

The results of network analysis indicate that *P. notoginseng* may exert therapeutic effects through genes such as IGF1R, EGFR, SRC, and JAK among its active components, aligning with existing research. Multiple studies have shown that IGF1R plays a crucial role in the development of DR. For example, one study demonstrated that transgenic mice overexpressing IGF1R exhibited retinal changes similar to those seen in human diabetic eye disease, including vascular alterations and neovascularization, even under normal glucose and insulin levels [32]. Additionally, IGF1R levels significantly increase with the progression of DR [33]. EGFR signaling is critical in the remodeling of the corneal stroma during diabetic conditions. Specifically, EGFR signaling influences the arrangement and distribution of collagen fibers and proteoglycans, thereby affecting corneal transparency and structural stability. Meanwhile, EGFR inhibitors show promise in preventing or treating corneal damage caused by diabetes. In studies on DR, the EGFR inhibitor AG1478 was found to inhibit inflammatory infiltration and neovascularization, reducing retinal dysfunction and structural damage in diabetic mice [34]. Similarly, the EGFR inhibitor erlotinib reduced pathological neovascularization in an oxygen-induced retinopathy (OIR) mouse model by inhibiting ADAM17 and EGFR [35]. These findings suggest that EGFR inhibition may protect retinal structure by reducing inflammation and neovascularization, potentially offering therapeutic benefits for diabetes-induced corneal damage. JAK2's role in ocular microvascular damage primarily involves its participation in the JAK-STAT signaling pathway, which promotes retinal ischemia-reperfusion injury. Expression of JAK2 and STAT3 proteins significantly increases in models of retinal ischemia-reperfusion injury, indicating the important role of the JAK-STAT pathway in retinal pathology [36]. In studies of oxygen-induced neovascularization in the mouse retina, activation of the JAK2/STAT3 signaling pathway was found to promote neovascularization. This further confirms JAK2's role in ocular microvascular damage, particularly in promoting neovascularization [37].

The results of enrichment analysis indicate that the therapeutic effects of *P. notoginseng* on diseases primarily occur through the advanced glycation end product (AGE)-RAGE, PI3-K/AKT, vascular endothelial growth factor (VEGF), and JAK-STAT pathways. Studies have shown that AGEs play a crucial role in retinal damage. These AGEs can interact with the receptor for AGEs (RAGE), leading to a series of molecular mechanisms that result in the loss of pericytes in the retina, induce inflammatory responses, and promote neovascularization [38]. Additionally, the binding of AGEs to RAGE activates intracellular signaling pathways, inducing oxidative stress, promoting inflammation and thrombosis, causing cell apoptosis, and enhancing the expression of VEGF, thereby triggering the formation of retinal neovascularization [39]. Indeed, VEGF is considered an important mediator of the pathophysiological changes associated with DR [40]. High levels of VEGF expression are positively correlated with the degree of microvascular damage in patients with DR, suggesting that VEGF may be involved in the damage process of microvascular structure and function in DR [29].

Molecular docking simulations revealed targets. Binding interactions between the active component quercetin in *P. notoginseng* and key targets such as SRC, IBF1R, JAK2, and EGFR. These proteins are involved in various cellular processes, including immune regulation, cell signaling, and regulation of cell proliferation and differentiation, which may contribute to *P. notoginseng*'s reparative effects on ocular microvasculature.

This study provides a comprehensive analysis of the potential targets and mechanisms of *P. notoginseng*, which can guide the development of new treatments for DR and DCs. However, it should be noted that the target data originate from databases, and the reliability and accuracy of the analysis and predictions depend on the quality of the data. Furthermore, additional in vivo and in vitro studies are needed to validate the findings and elucidate the specific roles of each component in *P. notoginseng*.

## 5. Conclusion

This study provides a robust scientific framework for evaluating the therapeutic effects of multi-component, multi-target active ingredients in the treatment of DR and DC using the principle of treating different diseases with the same therapy in *P. notoginseng*. By adopting an integrated approach that combines network pharmacology and molecular docking, we extensively investigated the chemical constituents of *P. notoginseng* and their potential biochemical processes involved in the treatment of DR and DC. Our findings highlight the complex interactions between active ingredients and multiple compounds and targets within the target gene network, thereby advancing our understanding of the potential therapeutic effects of *P. notoginseng*. Nevertheless, it is important to acknowledge the limitations of this study, *P. notoginseng* is generally safe at normal dosages and exhibits a range of pharmacological effects. However, high concentrations or prolonged use may lead to potential toxicity. Achieving sufficient concentrations of antioxidant components in retinal tissue through oral administration of *P. notoginseng* is challenging. To prevent harm to participants in future research, we conduct rigorous ethical reviews, obtain informed consent, and monitor and follow up with participants meticulously. Further research is needed to validate these findings regarding the plant components. This approach lays foundational groundwork for future investigations into the mechanisms of *P. notoginseng* in the treatment of DR and DC and underscores the potential applications of network pharmacology in drug design.

## Figures and Tables

**Table 1:** The names of the software/web tool/database used in this article.

Sr. no	Software/webtool/database name	Url	References
1.	ETCM 1.0	http://www.tcmip.cn/ETCM/	[9]
2.	TCMSP	https://old.tcmsp-e.com/tcmsp.php	[12]
3.	Swiss target prediction	https://www.swisstargetprediction.ch/	[14]
4.	GeneCards	https://www.genecards.org/	[15]
5.	OMIM	https://www.omim.org/	[16]
6.	PubChem	https://pubchem.ncbi.nlm.nih.gov/	[10]
7.	Bioinformatics web tool	https://bioinformatics.com.cn/	[11]
8.	String	https://string-db.org/	[17]
9.	Cytoscape-3.9.2	https://Cytoscape.org/	[18]
10.	Metascape	http://Metascape.org/	[19]
11.	David	http://david.ncifcrf.gov/tools.jsp	[20]
12.	ShinyGo 0.80	http://bioinformatics.sdstate.edu/go/	[21]

**Table 2:** The compound structures and parameters of the pharmaceutically viable components of *P. notoginseng*.

No.	Compound	DL	OB%	Molecular structure
1	DFV	32.76	0.18	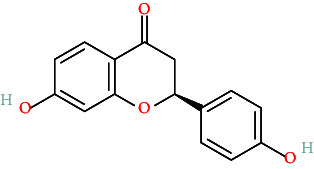
2	Mandenol	42	0.19	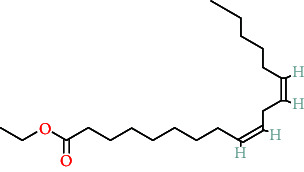
3	Ginsenoside f2	36.43	0.25	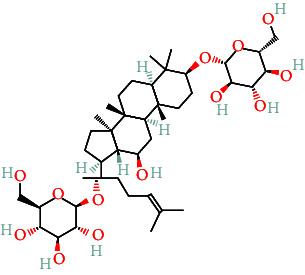
4	Quercetin	46.43	0.28	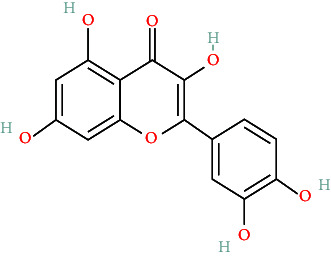
5	Diop	43.59	0.39	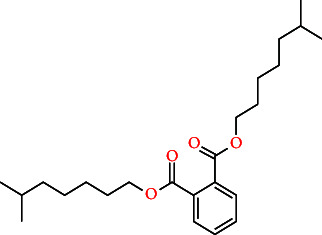
6	Ginsenoside rh2	36.32	0.56	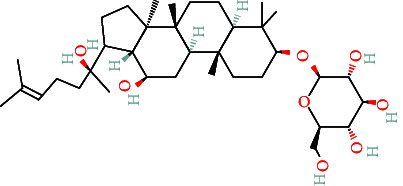
7	Beta-sitosterol	36.91	0.75	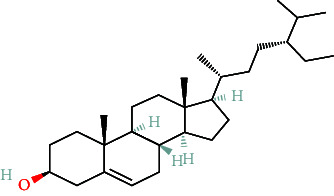
8	Stigmasterol	43.83	0.76	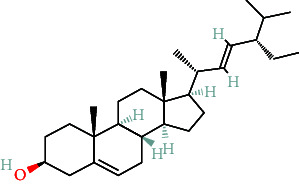

**Table 3:** Centrality measures for hub nodes.

Method	Equation	Evaluation of meaning
Degree centrality	$C_{D\left(V\right)=\frac{k_{v}}{\text{n}-1}}$	A node with high degree centrality has a greater number of direct connections
Closeness centrality	$C_{C\left(V\right)=\frac{n-1}{\sum_{u\neq v}d_{vu}}}$	A node with high closeness centrality can establish contact with other nodes in the network more rapidly
Betweenness centrality	$C_{B\left(V\right)=\sum_{s\neq v\neq t}\frac{\sigma_{st}}{\sigma_{st}}\left(v\right)}$	A node with high betweenness centrality serves as a bridge within the network, connecting different segments
Eigenvector centrality	$C_{E\left(V\right)=\frac{1}{\lambda }\sum_{u\in N\left(v\right)}\frac{C_{E}\left(U\right)}{k_{u}}}$	Eigenvector centrality considers the significance of a node's neighbors

**Table 4:** Topological parameters of protein–protein interaction (PPI).

No.	Gene	Degree	Eigenvector	Betweenness	Closeness
1.	ESR1	60.0	0.26029825	1830.5078	0.6666667
2.	EGFR	57.0	0.25677937	1412.401	0.64480877
3.	PTGS2	53.0	0.2180905	1408.2035	0.6344086
4.	SRC	48.0	0.23069571	829.0761	0.6145833
5.	PARP1	39.0	0.19917192	648.43427	0.5841584
6.	GSK3B	37.0	0.19160324	457.04306	0.59
7.	MAPK1	35.0	0.17568618	419.82135	0.57004833
8.	MMP2	34.0	0.17587411	311.58704	0.5539906
9.	KDR	31.0	0.17223382	286.46872	0.5488372
10.	ABL1	30.0	0.15419652	220.0955	0.53881276
11.	JAK2	29.0	0.15423055	231.14969	0.53881276
12.	CDK2	29.0	0.14399576	367.83554	0.5539906
13.	IGF1R	29.0	0.17529786	93.10595	0.5592417
14.	PGR	28.0	0.15608834	137.6011	0.5437788
15.	AR	27.0	0.15659745	143.44783	0.53636366
16.	HMGCR	27.0	0.08934841	519.34717	0.5339367
17.	CYP19A1	26.0	0.108166635	474.8154	0.5437788
18.	KIT	26.0	0.15391782	81.35702	0.5339367
19.	ABCB1	26.0	0.13954939	252.94966	0.5462963
20.	TERT	25.0	0.14361875	115.61944	0.529148
21.	MET	25.0	0.1536979	61.62283	0.5315315
22.	ABCG2	24.0	0.12147701	402.48206	0.52678573
23.	PRKCA	23.0	0.12550442	102.49683	0.52444446
24.	ESR2	22.0	0.11524542	237.0938	0.51754385
25.	CCNB1	22.0	0.13171011	162.74641	0.51754385
26.	CDK1	22.0	0.117557935	115.05387	0.51082253
27.	TOP2A	22.0	0.10495269	180.78407	0.5152838
28.	NR3C1	22.0	0.10344075	238.66478	0.51754385
29.	APP	21.0	0.11066522	249.47095	0.5315315
30.	PDGFRB	21.0	0.12370508	57.957745	0.5064378

**Table 5:** Topological parameters of fully connected network.

No.	Node	Degree	Eigenvector	Betweenness	Closeness
1.	DR DC	88.0	0.6381894	11555.384	0.7267442
2.	quercetin	19.0	0.08628814	1346.8325	0.44642857
3.	SRC	17.0	0.14355634	507.77997	0.4032258
4.	Mandenol	16.0	0.079092085	1005.76917	0.38819876
5.	AR	13.0	0.08808362	489.2598	0.36764705
6.	ABL1	11.0	0.087327406	193.99463	0.3633721
7.	ESR1	11.0	0.07723041	220.58867	0.3434066
8.	JAK2	11.0	0.080600314	209.80153	0.3633721
9.	IGF1R	11.0	0.0856934	182.22177	0.36764705
10.	KDR	9.0	0.074213035	185.4738	0.38109756
11.	PARP1	8.0	0.06409548	92.52725	0.3591954
12.	beta-sitosterol	7.0	0.029063523	105.171394	0.30339807
13.	Stigmasterol	7.0	0.029063523	105.171394	0.30339807
14.	PGR	7.0	0.058345072	71.59767	0.3511236
15.	CDK1	7.0	0.052697048	145.06108	0.37650603
16.	TERT	7.0	0.048327137	101.89296	0.35310733
17.	Panax Notoginseng	6.0	0.023246553	246.08841	0.37425148
18.	NR3C1	6.0	0.037145223	104.58414	0.349162
19.	GO:0060603	6.0	0.102378696	212.03792	0.4595588
20.	GO:0038083	6.0	0.10563588	136.20047	0.44964027
21.	CYP19A1	6.0	0.030232618	252.47372	0.37650603
22.	EGFR	6.0	0.046032853	74.72765	0.35511363
23.	ABCG2	6.0	0.04190826	73.86263	0.3511236
24.	CDK2	6.0	0.043788884	75.879684	0.35511363
25.	GO:0035173	5.0	0.08232467	179.28696	0.44642857
26.	ESR2	5.0	0.02840083	94.033646	0.35714287
27.	GO:0060444	5.0	0.09875435	136.0311	0.45620438
28.	GO:0051882	5.0	0.09898467	89.64235	0.44642857
29.	PDGFRB	5.0	0.03672219	56.195457	0.3434066
30.	CCNB1	5.0	0.036489718	44.62582	0.349162

**Table 6:** The results of molecular docking.

No.	Receptor	PDB ID	Ligand	Resolution	Binding energy
1.	SRC	4BVM	Quercetin	0.93 A	−8.16
2.	IBF1R	1P4O	Quercetin	1.5 A	−5.96
3.	JAK2	7TEU	Quercetin	1.45 A	−6.06
4.	EGFR	8A27	Quercetin	1.07 A	−6.29

## Data Availability

The data used to support the findings of this study are available from the corresponding author upon request.

## Supporting Information

Supporting Information 1 Figure S1: Protein-Protein Interaction (PPI) network diagram. The nodes in the diagram represent individual proteins. Each node is labeled with a unique identifier. The lines connecting the nodes in the diagram represent known interactions between proteins. These interactions can be physical associations, such as binding, or functional relationships, such as co-regulation.

Supporting Information 2 Table S1: The chemical components of Panax. notoginseng.
