# Supplementary figures and images for: Network Pharmacology Study on the Mechanisms of Panax Notoginseng in the Treatment of Diabetic Retinopathy and Cataract
Source: Appl Bionics Biomech. 2025 May 11;2025:6687606. doi: 10.1155/abb/6687606 (PMC12086034; doi:10.1155/abb/6687606)

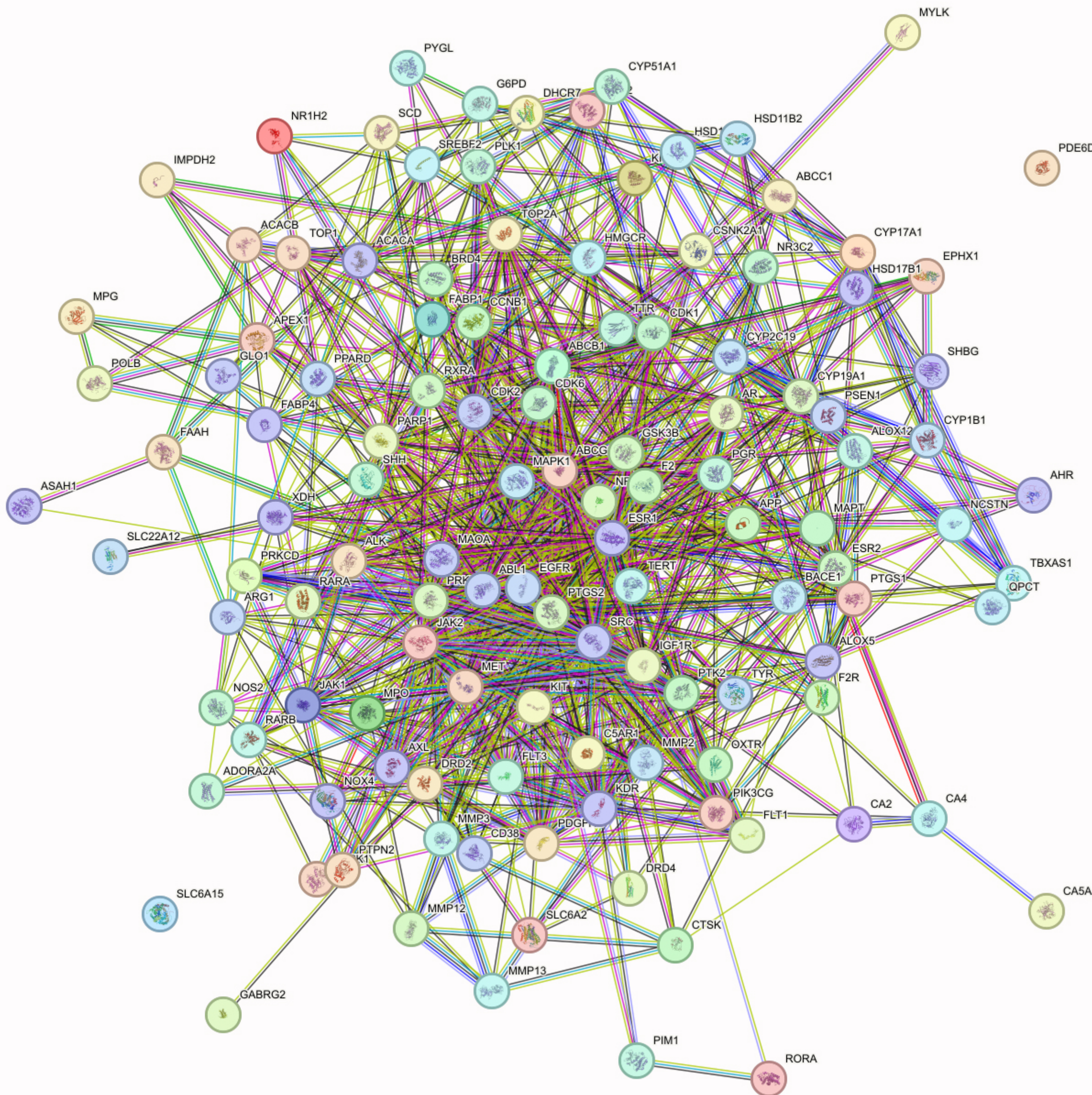

Supplement: Supporting Information 1 — Figure S1: Protein-Protein Interaction (PPI) network diagram. The nodes in the diagram represent individual proteins. Each node is labeled with a unique identifier. The lines connecting the nodes in the diagram represent known interactions between proteins. These interactions can be physical associations, such as binding, or functional relationships, such as co-regulation. [file 6687606.f1.pdf]
