# Supplementary material for: Network Pharmacology Study on the Mechanisms of Panax Notoginseng in the Treatment of Diabetic Retinopathy and Cataract
Source: Appl Bionics Biomech. 2025 May 11;2025:6687606. doi: 10.1155/abb/6687606 (PMC12086034; doi:10.1155/abb/6687606)
Supplement: Supporting Information 2 — Table S1: The chemical components of Panax. notoginseng. [file 6687606.f2.docx]

| **NO.** | **compound** |
| --- | --- |
| 1 | PEL |
| 2 | 2-ACETYLPYRROLE |
| 3 | (+)-Ledol |
| 4 | myristic acid |
| 5 | Oktadekan |
| 6 | Mandenol |
| 7 | Daturic acid |
| 8 | Hypnon |
| 9 | alpha-Guaiene |
| 10 | zoomaric acid |
| 11 | DFV |
| 12 | Cadalin |
| 13 | METHYL ISOPALMITATE |
| 14 | ZINC02169908 |
| 15 | hexanoic acid |
| 16 | 1H-Cycloprop(e)azulen-7-ol, decahydro-1,1,7-trimethyl-4-methylene-, (1aR-(1aalpha,4aalpha,7beta,7abeta,7balpha))- |
| 17 | PTL |
| 18 | farnesol |
| 19 | 3691-11-0 |
| 20 | oleanolic acid |
| 21 | Hexenal |
| 22 | Picein |
| 23 | Piceol |
| 24 | butylated hydroxytoluene |
| 25 | Diop |
| 26 | caprylic acid |
| 27 | lauric acid |
| 28 | (1S,4S)-7-isopropylidene-1,4-dimethyl-2,3,4,5,6,8-hexahydro-1H-azulene |
| 29 | Sitogluside |
| 30 | beta-sitosterol |
| 31 | DL-Glucuronic acid |
| 32 | 2-Coumarate |
| 33 | 5-METHYLFURFURAL |
| 34 | MEHQ |
| 35 | Stigmasterol |
| 36 | 2-octanone |
| 37 | TMH |
| 38 | (3S,5R,6S,8R,9R,10R,12R,13R,14R,17S)-17-[(2S)-2-hydroxy-6-methylhept-5-en-2-yl]-4,4,8,10,14-pentamethyl-2,3,5,6,7,9,11,12,13,15,16,17-dodecahydro-1H-cyclopenta[a]phenanthrene-3,6,12-triol |
| 39 | Ditertbutyl phthalate |
| 40 | (3S,5R,8R,9R,10R,12R,13R,14R,17S)-17-[(2S)-2-hydroxy-6-methylhept-5-en-2-yl]-4,4,8,10,14-pentamethyl-2,3,5,6,7,9,11,12,13,15,16,17-dodecahydro-1H-cyclopenta[a]phenanthrene-3,12-diol |
| 41 | (2S,3R,4S,5S,6R)-2-[(2S)-2-[(3S,5R,8R,9R,10R,12R,13R,14R,17S)-3-[(2R,3R,4S,5S,6R)-4,5-dihydroxy-6-(hydroxymethyl)-3-[(2S,3R,4S,5S,6R)-3,4,5-trihydroxy-6-(hydroxymethyl)oxan-2-yl]oxyoxan-2-yl]oxy-12-hydroxy-4,4,8,10,14-pentamethyl-2,3,5,6,7,9,11,12,13,15,1 |
| 42 | Ginsenoside Re |
| 43 | (3S,5R,6S,8R,9R,10R,12R,13R,14R,17S)-17-[(2R)-2-hydroxy-6-methylhept-5-en-2-yl]-4,4,8,10,14-pentamethyl-2,3,5,6,7,9,11,12,13,15,16,17-dodecahydro-1H-cyclopenta[a]phenanthrene-3,6,12-triol |
| 44 | Sanchinoside C1 |
| 45 | ginsenoside rh2 |
| 46 | beta-elemene |
| 47 | Linolenyl alcohol |
| 48 | 1-Hydroxycumene |
| 49 | (-)-alpha-cedrene |
| 50 | alloaromadedrene |
| 51 | hexanal |
| 52 | Gypenoside XIV_qt |
| 53 | oleic acid |
| 54 | palmitic acid |
| 55 | WLN: VH6 |
| 56 | 2,4-Heptdienal |
| 57 | [(1R)-1-methoxyethyl]benzene |
| 58 | Cyclooctadiene |
| 59 | 1,2-DIHYDRO-1,5,8-TRIMETHYLNAPHTHALENE |
| 60 | 1,4,6-trimethyl-1,2,3,4-tetralin |
| 61 | (1R,2S)-1-ethyl-2-methylcyclopropane |
| 62 | 1-methyl-5-isopropenyl cyclohexene |
| 63 | 10-Methylnonadecane |
| 64 | DICHLOROANILINE |
| 65 | 2,6-dimethyl-cyclohexanol |
| 66 | 3,5-Dimethoxyacetophenone |
| 67 | 3,4-dichloroaniline |
| 68 | (2E)-3-ethylpenta-2,4-dien-1-ol |
| 69 | Octadecyne |
| 70 | (9Z,12E)-octadeca-9,12-dienoic acid methyl ester |
| 71 | N3-Oxalyl-L-2,3-diaminopropanoate |
| 72 | (2R,3S,4S,5R,6S)-2-(hydroxymethyl)-6-[(2S)-6-methyl-2-[(3S,5R,6S,8R,9R,10R,12R,13R,14R,17S)-3,6,12-trihydroxy-4,4,8,10,14-pentamethyl-2,3,5,6,7,9,11,12,13,15,16,17-dodecahydro-1H-cyclopenta[a]phenanthren-17-yl]hept-5-en-2-yl]oxyoxane-3,4,5-triol |
| 73 | ginsenoside f2 |
| 74 | ginsenoside rb1 |
| 75 | (2S,3R,4S,5S,6R)-2-[(2S)-2-[(3S,5R,8R,9R,10R,12R,13R,14R,17S)-3-[(2R,3R,4S,5S,6R)-4,5-dihydroxy-6-(hydroxymethyl)-3-[(2S,3R,4S,5S,6R)-3,4,5-trihydroxy-6-(hydroxymethyl)oxan-2-yl]oxyoxan-2-yl]oxy-12-hydroxy-4,4,8,10,14-pentamethyl-2,3,5,6,7,9,11,12,13,15,1 |
| 76 | ginsenoside rb3 |
| 77 | ginsenoside rc |
| 78 | ginsenoside rd_qt |
| 79 | ginsenoside rf |
| 80 | (2S,3R,4R,5R,6S)-2-[[(2R,3R,4S,5S,6R)-2-[[(3S,5R,6S,8R,9R,10R,12R,13R,14R,17S)-3,12-dihydroxy-17-[(1S)-1-hydroxy-1,5-dimethylhex-4-enyl]-4,4,8,10,14-pentamethyl-2,3,5,6,7,9,11,12,13,15,16,17-dodecahydro-1H-cyclopenta[a]phenanthren-6-yl]oxy]-4,5-dihydroxy- |
| 81 | ginsenoside rg3 |
| 82 | (2R,3R,4S,5S,6R)-2-[[(3S,5R,6S,8R,9R,10R,12R,13R,14R,17S)-3,12-dihydroxy-17-[(2S)-2-hydroxy-6-methylhept-5-en-2-yl]-4,4,8,10,14-pentamethyl-2,3,5,6,7,9,11,12,13,15,16,17-dodecahydro-1H-cyclopenta[a]phenanthren-6-yl]oxy]-6-(hydroxymethyl)oxane-3,4,5-triol |
| 83 | (2S,3S,4S,5R,6R)-6-[[(3S,4aR,6aR,6bS,8aS,12aS,14aR,14bR)-4,4,6a,6b,11,11,14b-heptamethyl-8a-[oxo-[[(2S,3R,4S,5S,6R)-3,4,5-trihydroxy-6-(hydroxymethyl)-2-tetrahydropyranyl]oxy]methyl]-1,2,3,4a,5,6,7,8,9,10,12,12a,14,14a-tetradecahydropicen-3-yl]oxy]-3,4-di |
| 84 | ZINC01532096 |
| 85 | notoginsenosider1 |
| 86 | notoginsenosider2 |
| 87 | notoginsenosider3 |
| 88 | notoginsenosider3_qt |
| 89 | notoginsenosider4 |
| 90 | Butylcyclobutane |
| 91 | (1S,4S,4aS,6S,8aS)-4-isopropyl-1,6-dimethyldecahydronaphthalene |
| 92 | gypenosideix |
| 93 | gypenosidexvii |
| 94 | isopulegone |
| 95 | NaPst |
| 96 | 10Z,13Z-nonadecadienoic acid |
| 97 | Lutein |
| 98 | panaxatriol |
| 99 | panaxydol |
| 100 | NSC692928 |
| 101 | panaxytriol |
| 102 | NSC 308879 |
| 103 | WLN: QR DG |
| 104 | protopanoxadiol |
| 105 | sanchinan-a |
| 106 | α-cyperene |
| 107 | (4aS,9aR)-2,9,9-trimethyl-5-methylene-4,4a,6,7,8,9a-hexahydro-3H-benzo[7]annulene |
| 108 | α-copaene |
| 109 | (5S)-5-ethyloxolan-2-one |
| 110 | stearic acid |
| 111 | MYS |
| 112 | Heptadekan |
| 113 | Henicosane |
| 114 | methyl palmitate |
| 115 | ()-beta-Pinene |
| 116 | Germacrene D |
| 117 | Hepanal |
| 118 | Ethylpalmitate |
| 119 | quercetin |
